# Low-Dose Chidamide Treatment Displays Sex-Specific Differences in the 3xTg-AD Mouse

**DOI:** 10.3390/biom13091324

**Published:** 2023-08-29

**Authors:** Jessica Dennison, Armando Mendez, Angela Szeto, Ines Lohse, Claes Wahlestedt, Claude-Henry Volmar

**Affiliations:** 1 Department of Psychiatry & Behavioral Sciences, University of Miami Miller School of Medicine, Miami, FL 33136, USA; jxd1249@miami.edu (J.D.);; 2 Center for Therapeutic Innovation, University of Miami Miller School of Medicine, Miami, FL 33136, USA; 3 Diabetes Research Institute, Division of Endocrinology, Diabetes, and Metabolism, University of Miami Miller School of Medicine, Miami, FL 33136, USA

**Keywords:** Alzheimer’s, epigenetics, HDAC inhibitor, sex differences, 3xTg-AD mouse

## Abstract

Epigenetic compounds have become attractive small molecules for targeting the multifaceted aspects of Alzheimer’s disease (AD). Although AD disproportionately affects women, most of the current literature investigating epigenetic compounds for the treatment of AD do not report sex-specific results. This is remarkable because there is rising evidence that epigenetic compounds intrinsically affect males and females differently. This manuscript explores the sexual dimorphism observed after chronic, low-dose administration of a clinically relevant histone deacetylase inhibitor, chidamide (Tucidinostat), in the 3xTg-AD mouse model. We found that chidamide treatment significantly improves glucose tolerance and increases expression of glucose transporters in the brain of males. We also report a decrease in total tau in chidamide-treated mice. Differentially expressed genes in chidamide-treated mice were much greater in males than females. Genes involved in the neuroinflammatory pathway and amyloid processing pathway were mostly upregulated in chidamide-treated males while downregulated in chidamide-treated females. This work highlights the need for drug discovery projects to consider sex as a biological variable to facilitate translation.

## 1. Introduction

Regulation of gene transcription through modifiable epigenetic proteins has become an attractive therapeutic approach in many diseases including Alzheimer’s disease (AD). This is due to the multitarget approach that epigenetic compounds fundamentally offer. Epigenetic compounds modify the action of histone-interacting regulatory proteins that are involved in acetylation, phosphorylation, methylation, and ubiquitination of histone proteins. Histone acetylation is the most-studied histone modification in the context of AD as dysregulation of histone acetylation and increases in histone deacetylase (HDAC) activity have been correlated with worse AD pathological outcomes [1,2,3]. Inhibition of HDACs have shown potential as a therapeutic intervention for AD and are beginning to be explored clinically [4,5].

Female sex is a leading risk factor in the development of AD, second only to old age. Clinical data show that females are both more likely to develop AD and experience worse pathological outcomes [6,7,8]. Furthermore, there is sexual dimorphism seen in the response to clinical treatments [9,10]. However, sex differences in both pre-clinical studies and clinical trial outcomes are often under- or not reported. This has consequently created a gap in our understanding of both the disease and possible treatment avenues. The effects of epigenetic compounds in AD should especially be analyzed striated by sex due to males and females having differing baseline gene expressions, in addition to the unknown interactions between sex hormones and epigenetic modifiers [11]. Epigenetic compounds target a large number of mechanisms, including histone and non-histone protein modifications affecting gene expression and a number of biological pathways. This makes looking at sex even more critical while studying these compounds, especially given sex-specific differences in the epigenetic landscape [12].

Class I HDAC inhibitors have been implicated in numerous neuropsychiatric diseases including Parkinson’s, Huntington’s, and AD, yet sexual effects have not been explored in detail [13]. Here, we show one example of how an epigenetic compound, the class I HDAC inhibitor chidamide (tucidinostat) [14], can affect male and female mice in different, and sometimes opposing ways. Chidamide, which is in clinical trials in the U.S. for various cancers, is a benzamide class of HDAC inhibitors that has been demonstrated to inhibit class I HDACs 1, 2, 3, and class IIB HDAC10 in the low nanomolar range, and crosses the blood–brain barrier [15]. A recent study used a very low dose of chidamide (0.1 mg/kg) in a mouse model of immune thrombocytopenia and reported modulation of histone H3K27 acetylation occurred at this low dose [16]. HDAC inhibitors used in high doses cause some adverse effects, making the use of a chronic, low-dose an attractive treatment regimen [17].

Here, we report that administration of a very low dose of chidamide has sex- and brain-specific effects on gene expression and AD pathology. The data presented here support the hypothesis that epigenetic compounds can cause drastically opposing sex-specific effects on key therapeutic targets for Alzheimer’s disease and others.

## 2. Materials and Methods

### 2.1. HDAC Inhibitor Assay

HDAC Inhibitor Assays were performed by BPS Bioscience Inc. (San Diego, CA, USA). The effects of chidamide on the activities of recombinant HDAC1, HDAC2, HDAC3/NCOR2, and HDAC8 were tested using an enzymatic assay. SAHA (Cayman Chemicals; Ann Arbor, MI, USA) and TSA (Selleck Chemicals; Houston, TX, USA) were used as reference controls. The enzymatic reactions for the HDAC enzymes were conducted at 37 °C for 30 min. Fluorescence was measured at excitation 360 nm and emission 460 nm with a Tecan Infinite M1000 microplate reader.

### 2.2. Animals

Male and female triple-transgenic AD (3xTg-AD) [18] mice from the NIH-supported Mutant Mouse Resource & Research Centers (MMRRC) were purchased through the Jackson Laboratory (Farmington, CT, USA). Mice were housed four animals per cage in an AAALAC-accredited animal facility at the University of Miami Miller School of Medicine. All experiments were approved by the University of Miami Miller School of Medicine Institutional Animal Care and Use Committee (IACUC).

### 2.3. Treatment

Male and female mice (4–5 males and 5 females per treatment group) were treated by oral gavage with vehicle (saline, 5% Tween 80, 4.6% PEG 400) or 0.01 mg/kg Chidamide (Selleck Chemicals LLC; Houston, TX, USA) diluted in vehicle. Formulations were made fresh weekly and sonicated briefly to mix. Beginning at 4 months of age (prior to disease development), mice were treated 5 days a week for 9 months.

### 2.4. Behavior

A series of behavior tests intended to test motor coordination, anxiety, spatial learning, and reference memory was performed as previously performed by our lab [19]. Motor coordination was assessed at 11 months of age using rotarod. Anxiety was assessed by open field at 7 months and elevated O-maze at 11 months [20,21]. Spatial learning and reference memory were tested using Y-maze at 6 and 10-months old, Object Location Memory (OLM) at 10 months old, novel object recognition (NOR) [22], at 7 and 10 months old, and the Barnes Maze at 8 months old [23]. Video tracking was performed with the automated EthoVision XT software 15 (Noldus; Leesburg, VA, USA). Mice were habituated to the testing room for 1 h before testing commenced. Arenas were thoroughly cleaned with 70% ethanol between mice. Experimenters were blinded to treatment groups during acquisition and analysis. Further details of each test can be found in Supplementary Materials Part 1.

### 2.5. Glucose and Insulin Tolerance Testing

Mice underwent intraperitoneal (i.p.) glucose tolerance testing (GTT) at 10 months of age, and insulin tolerance testing (ITT) at 12 months of age [24]. For both tests, mice were food fasted 6 h prior to testing, with access to water. For GTT, mice were injected with 1.5 g/kg body weight glucose diluted in saline. For ITT, mice were injected with 0.8 U/kg body weight insulin. For both tests, blood glucose was measured from a tail prick by a glucometer (Bayer; Leverkusen, Germany) at 0, 15, 30, 60, 90, and 120 min.

### 2.6. Tissue Collection

At 13 months of age, mice were anaesthetized with isoflurane and euthanized via cardiac puncture. The brain was extracted and quickly dissected on ice, and snap-frozen in liquid nitrogen. The samples were stored at −80 °C until processed. For each mouse, brain regions from one hemisphere were used for RNA extraction and qPCR and sections from the other hemisphere were used for protein extraction and ELISAs.

### 2.7. ELISA

Protein lysates were prepared by sonicating tissue in M-PER Mammalian Protein Extraction Reagent (Thermo Fisher Scientific; Waltham, MA, USA) supplemented with protease and phosphatase inhibitor (Thermo Fisher Scientific). Total protein was quantified using Pierce BCA Protein Assay Kit (Thermo Fisher Scientific). Human Aβ_1–42_, Aβ_1–40_, total tau, phosphorylated-tau Serine-396, and phosphorylated-tau Threonine-181 were measured in brain tissue by enzyme-linked immunosorbent assay (ELISA; Invitrogen, Thermo Fisher Scientific), and measured using the EnVision^®^ multimode plate reader (Perkin Elmer; Waltham, MA, USA).

### 2.8. RNA Analysis

RNA was isolated from tissue using TRIzol^®^ reagent (Life Technologies, Thermo Fisher Scientific) and a Qiagen RNeasy^®^ Mini Kit (Qiagen; Hilden, Germany). RNA concentrations and quality were determined using NanoDrop 2000 UV-Vis Spectrophotometer (Thermo Fisher Scientific). cDNA was created using qScript cDNA Synthesis kit (Quantabio; Beverly, MA, USA). RT-qPCR was run using TaqMan 2-step RT-qPCR reagents (Thermo Fisher Scientific) on the QuantStudio Flex RT-qPCR system (Applied Biosystems, Thermo Fisher Scientific). Fold-change in gene expression relative to non-transduced controls were normalized. Results presented are based on fold-change using the 2^(−ΔΔCt)^ method.

The NanoString Technologies nCounter^®^ Mouse Neuropathology Panel was used to assess ERC RNA. This panel screens expression of 770 genes specific for neurodegeneration. We also included a custom 30-gene spike-in code set to include specific genes of interest. Assays were performed using the NanoString protocols, and data were normalized in nSolver^®^ analysis software v 2.0.

NanoString nCounter^®^ Advanced Analysis was used to identify differentially expressed genes (DEGs) which were considered significant if they had a Q value < 0.01. Heatmaps were created with Morpheus Software (accessed on 20 February 2022; software.broadinstitute.org/Morpheus). Ingenuity Pathway Analysis (Qiagen) was used to run comparison analyses of differentially regulated pathways between male and female animals.

### 2.9. Statistics

Data are expressed as the mean ± SEM. Statistical analyses and graphing were performed with GraphPad Prism 7 (GraphPad Software; San Diego, CA, USA). Unpaired Student’s *t*-test or Mann–Whitney test (where appropriate) was used for comparisons of two means. One-way ANOVA with Tukey’s or Dunnett’s post-hoc analysis was used for multiple comparisons when more than two means were being compared. Repeated measures ANOVA was used to analyze the Barnes maze and open field where the same mice were measured over time. Tukey’s or Dunnett’s adjusted *p* values are presented. Adjusted *p* < 0.05 was deemed to be of statistical significance. Outliers were determined with Grubbs’ test, alpha = 0.05. 

## 3. Results

### 3.1. Inhibitory Effects of Chidamide on Class I HDAC Activity

The IC_50_ of chidamide against the class I HDACs (HDAC1, HDAC2, and HDAC3/NCOR2), were 0.25, 0.82, and 1.0 μM, respectively (Figure S1). Our results were slightly different from previously reported IC_50_ values for this compound which reported highest inhibition of HDAC3 at 67 nM [14]. This difference is most likely due to experimental conditions: for our study the reactions were conducted at 37 °C for 30 min; in Ning et al. the reaction was carried out at room temperature for 17 h. For reference, the IC_50_ of SAHA against these were 0.049, 0.082, and 0.052 μM, respectively. The IC_50_ of Chidamide for the class I HDAC, HDAC8, was greater than 10 μM, or no significant inhibition was detected at 10 μM. For reference, the IC_50_ of TSA against HDAC8 was 1.3 μM.

The selective inhibition observed with chidamide against class I HDACs is similar to that of other benzamide class HDAC inhibitors. For example, Entinostat (MS-275), which is currently in clinical trials for cancer treatment, selectively inhibits HDAC1, HDAC2, and HDAC3 with an IC_50_ of 0.54, 0.61, and 0.62 μM, respectively [25]. However, Entinostat is reported to inhibit HDAC8 at an IC_50_ of 9.88 μM. Tacedinaline (CI-994) also selectively inhibits HDAC1, HDAC2, and HDAC3 at an IC_50_ of 0.9, 0.9, and 1.2 μM, respectively, and is not reported to inhibit HDAC8 [26]. 

### 3.2. Behavioral Assessment of 3xTg-AD Mice after Chidamide Treatment

To investigate the effect of chronic, low-dose chidamide treatment on AD-related cognitive deficits, male and female 3xTg-AD mice underwent a battery of behavioral tests to assess anxiety, learning, and memory (Figure S2). A difference was observed in 10-month-old, treated males in NOR. Chidamide-treated males spent a significantly (*t*_(6)_ = 6.14, *p* < 0.001) longer time exploring the novel object compared to the familiar object than did vehicle males. However, the low recognition index of the vehicle males also suggests neophobia, or fear of the new objects, a phenomenon that is not clearly understood in mouse models [27]. Overall, the behavior data do not reach the appropriate effect size to distinguish any meaningful results.

### 3.3. Chidamide-Treated Males Show Improved Glucose Tolerance

**Figure 1:**
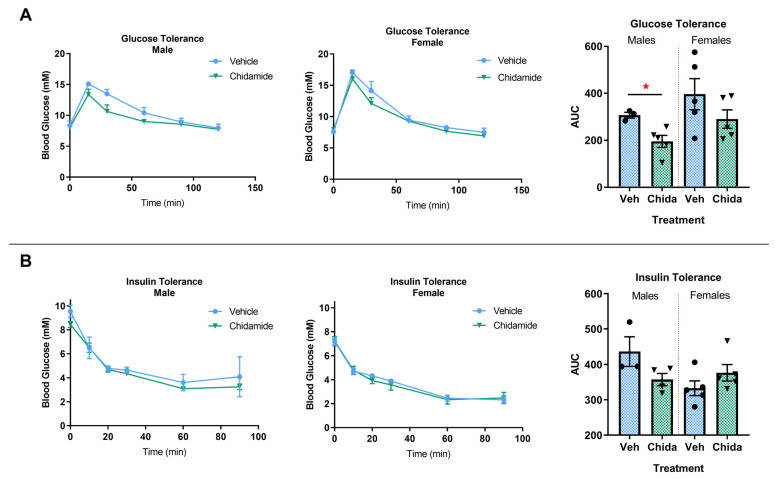
Glucose tolerance is significantly improved in chidamide-treated males. (**A**) Glucose tolerance testing in males and females at 10 months of age. (**B**) Insulin tolerance testing in males and females at 12 months of age. The graphs represent mean ± SEM. Unpaired Student’s *t*-test or Mann–Whitney test was used for comparisons of two means; * *p* < 0.05; *n* = 3–5/group.

The 3xTg-AD model has been reported to have decreased tolerance to glucose at 10 months of age compared to wild-type mice, which may exacerbate AD pathology [28]. We measured glucose tolerance at 10 months of age, the age that Vandal et al. began observing significant disfunction in the 3xTg-AD mice [28], and saw a significant improvement in glucose tolerance in chidamide-treated males (*t*_(6)_ = 3.15, *p* < 0.05; Figure 1A). Chidamide-treated females showed a trend towards improved glucose tolerance (*t*_(8)_ = 1.38, *p* = 0.21), but this did not reach significance (Figure 1A). Of note, females displayed greater variability in glucose tolerances, with AUC ranging from 200 to nearly 600, which may be due to hormonal variations [29]. A recent study reported female mice to be more glucose-tolerant than males and less sensitive to blood glucose fluctuations than males given various stressors [29]. No significant differences were seen in insulin tolerance with chidamide treatment at 12 months, in males (*t*_(6)_ = 1.37, *p* = 0.22) or females (*t*_(7)_ = 0.88, *p* = 0.41) (Figure 1B).

### 3.4. AD-like Pathology Displays Sex-, Brain Region-, and Treatment-Specific Differences

**Figure 2:**
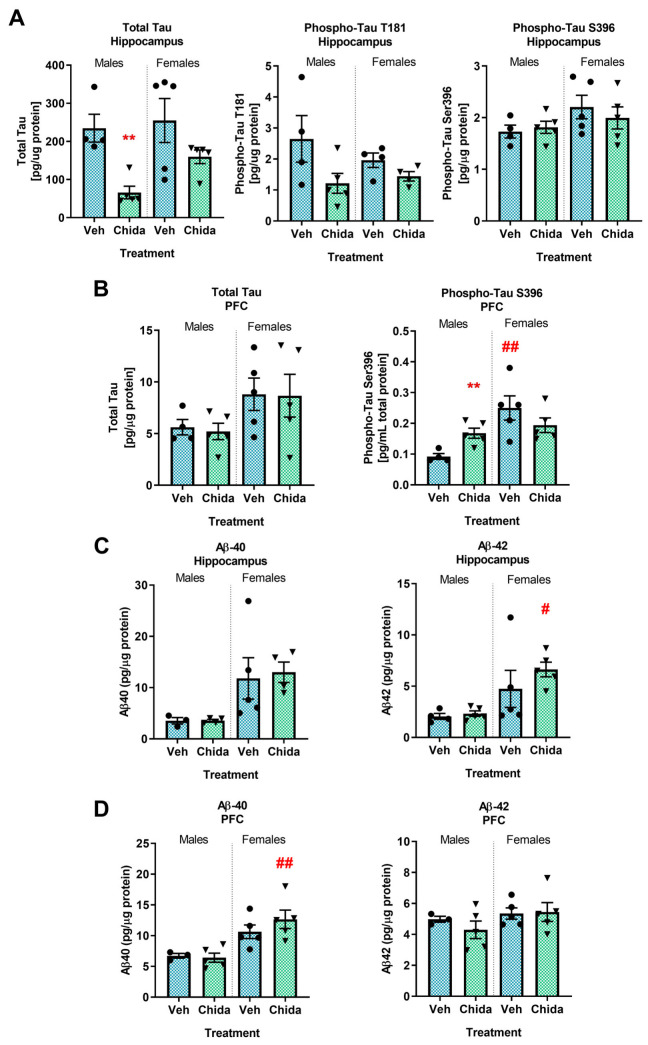
AD pathological hallmarks show sex-, treatment- and brain region-dependent differences. Levels of total tau and phosphorylated tau species in the (**A**) hippocampus and (**B**) PFC. Levels of Aβ-40 and -42 in the (**C**) hippocampus and (**D**) PFC. The graphs represent mean ± SEM. Unpaired Student’s *t*-test or Mann–Whitney test was used for comparison between treatments within sex. Tukey’s multiple comparisons test was used for comparison between sexes within treatment groups. ** *p* < 0.01 for significance between treatment groups within sex; # *p* < 0.05, ## *p* < 0.01 for significance between sex within treatment groups; *n* = 3–5/group.

To assess the effect of chronic, low-dose chidamide on AD-like pathology in vivo, ELISA for tau and Aβ species was performed using hippocampal and pre-frontal cortex (PFC) tissue (Figure 2). In the hippocampus (Figure 2A), chidamide-treated males had significantly lower levels of total tau compared to vehicle males (*t*_(7)_ = 4.56, *p* < 0.01). Chidamide-treated females did not show a significant difference in total tau levels compared to vehicles (*t*_(4.76)_ = 1.58, *p* = 0.18) due to the significant variance in vehicle total tau distribution (F_(4,4)_ = 10.49, *p* < 0.05). Tau species phosphorylated at threonine 181 (T181) showed a trend for decreased levels with chidamide treatment in both males (*t*_(7)_ = 1.9, *p* = 0.099) and females (*t*_(6)_ = 1.85, *p* = 0.11). In PFC (Figure 2B), levels of tau species phosphorylated at residue serine 396 (S396) were significantly greater in male chidamide-treated compared to vehicle (*t*_(6)_ = 3.96, *p* < 0.01). While there was no significant difference in females between treatment groups (*t*_(7)_ = 1.22, *p* = 0.26), there was a greater level of phospho-tau S396 in vehicle females compared to vehicle males (*p* < 0.01). 

Levels of both amyloidogenic (Aβ-42) and non-amyloidogenic (Aβ-40) Aβ species were also measured with ELISA in the hippocampus and PFC. Levels of Aβ-40 were greater in chidamide-treated females compared to males in PFC (*p* < 0.01). Aβ-42 was significantly greater in the hippocampus of female- compared to male-treated mice as tested by Tukey’s multiple comparisons test (*p* < 0.05).

### 3.5. Chidamide Treatment Affected Gene Expression More in Male Brains

**Figure 3:**
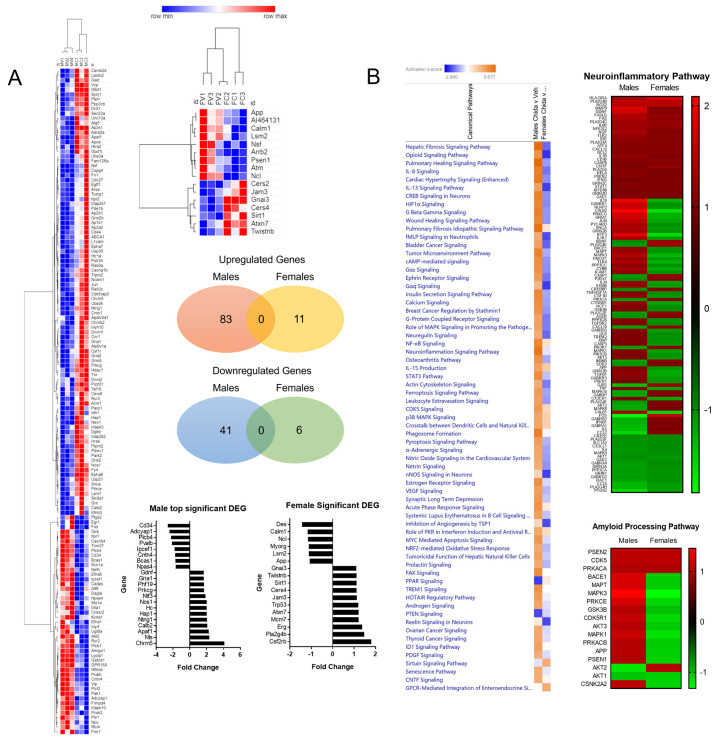
Significant differentially expressed genes between chidamide and vehicle separated by sex (**A**). Males showed greater DEGs in response to chidamide treatment than females. Pathway analysis shows a majority of pathways, including the neuroinflammatory pathway and amyloid processing pathway, are upregulated in male chidamide-treated mice compared to vehicle-treated males, while a majority of pathways are downregulated in female chidamide-treated mice compared to vehicle-treated females (**B**). NanoString data analyzed using nSolver Analysis Software 4.0; heatmaps created with Morpheus Software.

NanoString Neuropathology Panel was used to determine differentially expressed genes in chidamide versus vehicle animals (Figure 3A). As expected, chidamide affected transcription of genes; however, these transcriptional changes occurred at different magnitudes between male and female mice. Male chidamide-treated animals had 124 significant differentially expressed genes (DEG), 83 were increased and 41 were decreased, compared to vehicle. In contrast, female chidamide-treated animals had 17 DEGs, 11 were increased and 6 were decreased, compared to vehicle controls. Furthermore, Ingenuity Pathway Analysis revealed many disease-relevant pathways to be differentially regulated in chidamide-treated mice, and that the relationship is sex dependent (Figure 3B). Most pathways, including the neuroinflammation signaling pathway and amyloid processing pathway, were upregulated in males treated with chidamide compared to vehicle and downregulated in females treated with chidamide compared to vehicle. 

**Figure 4:**
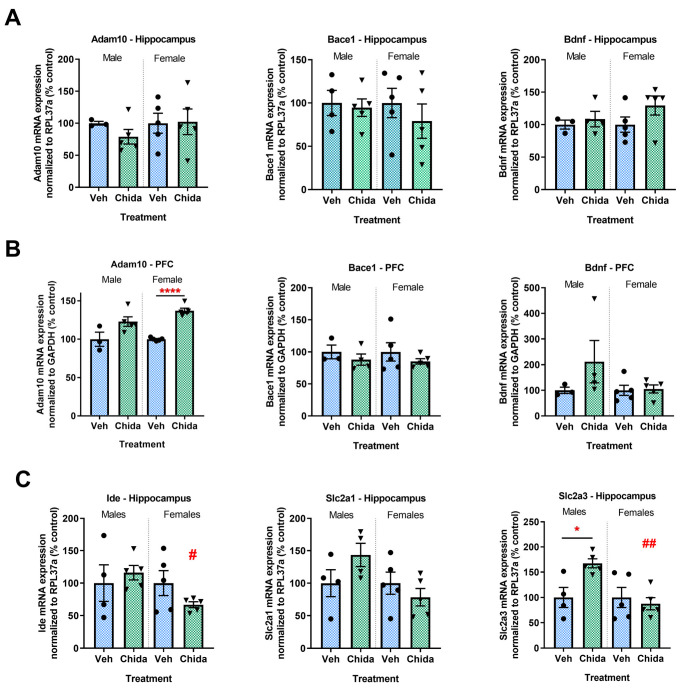
Long-term, low-dose chidamide treatment shows differing and sometimes opposing sex-dependent responses in AD- and metabolism-related gene expression. AD-related genes in the (**A**) hippocampus and (**B**) PFC. (**C**) Glucose transport genes in the hippocampus. The graphs represent mean ± SEM. Unpaired Student’s *t*-test or Mann–Whitney test was used for comparisons of two means. Tukey’s or Dunnett’s multiple comparisons test was used for comparison between sexes within treatment groups. * *p* < 0.05, **** *p* < 0.0001 for significance between treatment groups within sex; # *p* < 0.05, ## *p* < 0.01 for significance between sex within treatment groups; *n* = 3–5/group.

A select number of genes related to AD (*Adam10*, *Bace1*, *Bdnf*) or cellular glucose metabolism (*Ide*, *Slc2a1*, *Slc2a3*) were analyzed by qPCR (Figure 4). *Adam10*, a gene involved in non-amyloidogenic processing of amyloid precursor protein (APP) was significantly greater in chidamide-treated females (*t*_(8)_ = 10.94, *p* < 0.0001), and showed a trend for increase in chidamide-treated males (*t*_(6)_ = 2.16, *p* = 0.074) in the PFC, while no difference was observed in the hippocampus. Chidamide-treated males showed a significant increase in the gene that encodes GLUT3 (*Slc2a3*; *t*_(4)_ = 3.1, *p* < 0.05) which is responsible for glucose transport into neurons, and a trend for an increase in *Slc2a1* (*t*_(6)_ = 1.6, *p* = 0.16), which encodes GLUT1 which is responsible for glucose transport from blood into the brain.

## 4. Discussion

Decreased histone acetylation, especially due to increases in the class I HDACs, HDAC2 and HDAC3, has been associated with decreases in cognition and increases in AD pathology [30,31,32,33]. Inhibition of certain HDACs has hence become of therapeutic interest for AD. This is the first study to use a very low dose of the HDAC inhibitor chidamide chronically in a rodent model of AD, and is the first study to look at the effects of chidamide on the brain. Typical doses being used in cancer studies range from 10 to 50 mg/kg [14,34], while here we used 0.01 mg/kg given chronically. For the length of treatment used in this study, chronic, low-dose chidamide does not appear to significantly alter progression of AD-like pathology in this mouse model, despite transcriptomic changes. We initially set out to look at the effect of low-dose chidamide on AD but found that there were major sex differences that skewed the data, and masked many of the sex-dependent effects of the treatment, resulting in low *n* per treatment group. Notably, prior studies looking at HDAC inhibition in the 3xTg-AD mouse have used only males or have not specified sex [30,35,36,37]. Despite our low power, we still observed some statistically significant differences resulting from our low-dose treatment. The present study displays transcriptomic differences in response to an HDAC inhibitor between male and female 3xTg-AD mice. 

Previous studies using 3xTg-AD mice have reported improvements in Morris Water Maze and Barnes Maze performance with HDAC inhibitor treatment [30,35,36,38], and either improvement or no change in NOR [30,36]. The effects of HDAC inhibitors on cognition in AD mouse models has been varied, with some studies reporting no effect at all [39], and others showing significant improvement in behavioral deficits [30,36,37,38,40]. The present study was too underpowered to detect meaningful differences in behavior given the variability in performance. A long-term study with more animals should be considered in the future.

Dysregulation of glucose metabolism shares a strong relationship with AD progression; therefore, we wanted to look at the effect of chidamide treatment on glucose metabolism. Here, we report improved glucose tolerance only in chidamide-treated males, as well as increases in mRNA expression for the glucose transporters GLUT1 and GLUT3 within the brain. GLUT1 which is responsible for the transport of glucose across the blood–brain barrier, and GLUT3 which facilitates the transport of glucose into neurons, have both been found to be lowered in the brains of AD patients compared to healthy controls, and most likely contribute to the deficits in brain glucose metabolism seen in AD patients [41,42]. Female mice have been reported to be more glucose-tolerant than males and less sensitive to blood glucose fluctuations than males given various stressors, which may contribute to the lack of response seen in females [29].

We observe that treatment with chidamide can have different positive effects in the context of AD based on sex and brain region. An increase in *Adam10* expression, which was significant in the PFC of chidamide-treated females, is important in promoting anti-amyloidogenic APP processing, possibly helping to slow progression of AD pathology. However, a corresponding decrease in PFC Aβ-42 was not observed. The lack of change in Aβ-42 levels may be the reason there is no difference in behavior with treatment.

In the present study, mice treated with chidamide showed a reduction in total tau in the hippocampus. It has previously been reported that reduction in endogenous tau in hAPP mice prevented behavioral deficits and protected against excitotoxicity without altering Aβ levels [43], and that reducing levels of total tau lessened behavioral deficits even though neurofibrillary tangles continued to accumulate [44]. In our case, we did not see a difference in behavior despite the decrease in total tau, perhaps due to the lack of change in Aβ. With regards to phosphorylated tau species, we saw a decrease in phospho-tau at T181 but an increase in phospho-tau at S396. Increases in phosphorylated tau at T181 have been described as an early biomarker in AD [45], suggesting that chidamide can cause amelioration of AD-like pathology at this residue. An increase in phosphorylation at site S396 could indicate an increase in the protein kinase p38γ, which has been demonstrated to be protective against excitotoxicity [46]. Additionally, phosphorylation at S396 is reportedly required for long-term depression (LTD) in the hippocampus [47]. One study identifies tau phosphorylation at S396-404 to be an early event in AD [48]; however, another study using human brain tissue found S396 tau to have high variability in brain tissue and to only show minor progression in disease compared to healthy brains [49].

Perhaps the most striking differences between males and females are represented by the gene expression and pathway analysis data. Male chidamide-treated mice had seven times more significantly differentially regulated genes in the entorhinal cortex than did females, and pathway analysis showed that chidamide treatment had opposite directional effects in males compared to females. Treated males displayed increased activation and females decreased activation of pathways involved in CREB signaling, cAMP-mediated signaling, insulin secretion signaling, neuroinflammation signaling, and APP processing. Sex differences in immune response have been documented through the years but have been less studied in the context of the brain. Recent studies have observed sex-specific immune signatures in adult mouse microglia. These immune signatures remained even when transplanted into the brains of the opposite sex, demonstrating strong genetic and epigenetic regulation of microglia response [50]. The relationship between sex and neuroinflammation is largely affected by age, as recently reported, with females having a greater neuro-inflammatory response to LPS when aged, compared to males [51].

Others have also observed opposing epigenetic responses in male and female mice. Tyler et al. report opposite acetylation changes in response to arsenic exposure in the brains of C57Bl/6 mice, H3K9 acetylation increased in male and decreased in females [52]. These results are not unexpected given that nuclear hormone receptors regulate gene transcription through the recruitment of co-activators and/or co-repressors to specific sites on the DNA (hormone response elements) [53]. The sex differences in response to chidamide treatment may be due to a number of additional variables including baseline differences in these mice, as previously discussed [54], sex differences in drug metabolism, interaction with the immune system, different compensatory mechanisms, and more. We cannot fully understand the interaction between sex and chidamide in this study, as it is surely a complex one, but want to highlight that there is a strong interaction and that this should be considered in all drug studies, especially those utilizing epigenetic regulating compounds.

The data presented here, and in other, similar studies that analyze data striated by sex, suggest that not paying attention to gender differences is distorting clinical trial outcomes as well as the pipeline to clinical trials. In order to close the gap between preclinical and clinical research, sexual dimorphism in disease and treatment response should be taken into account and would likely lead to more consistent results. This is not limited to AD therapeutics, but to most neurodegenerative and multifaceted diseases. It begs the question as to whether we need different biomarkers depending on sex or if we should be looking at dosage and timing of therapeutics differently based on sex. It should be recognized that, despite the very low dose and the limitations of our sample size, we still had large differences in drug response between sexes. In some cases, we even saw opposite effects, which if combined would cancel out any response if males and females were analyzed together. This has implications across drug development in every field, especially if a negative or positive effect is being overlooked due to improper data handling.

## Data Availability

The data presented in this study are available on request from the corresponding author.

## Supplementary Materials

The following supporting information can be downloaded at: https://www.mdpi.com/article/10.3390/biom13091324/s1, Part 1. Supplementary Methods; Figure S1: IC50 for chidamide; Figure S2: Sensorimotor, anxiety-related, and memory-related behavior. The graphs represent mean ± SEM. Unpaired Student’s *t* test or Mann–Whitney test was used for comparisons of two means. *** *p* < 0.001; n = 3–5/group.
