# Supplementary material for: Low-Dose Chidamide Treatment Displays Sex-Specific Differences in the 3xTg-AD Mouse
Source: Biomolecules. 2023 Aug 29;13(9):1324. doi: 10.3390/biom13091324 (PMC10526199; doi:10.3390/biom13091324)
Supplement: Supplementary file 1 [file biomolecules-13-01324-s001.zip › biomolecules-2476970-supplementary.pdf]

Part 1. Supplementary Methods

1. Rotarod: The Harvard Apparatus Rota Rod was used to test motor coordination. The rotarod treadmill was set at a steady start speed of 1-4 rpm. Acceleration started 3 seconds after placing the mouse on the rod. Three trials were performed with 30 min inter-trial intervals (ITI). Motor coordination was assessed by the latency to fall on the very first trial between treatment groups.
2. Elevated O-Maze: The elevated O-maze consists of an elevated ring-shaped track with walls on two quarters, and no walls on the other two quarters. Mice are given 5 min to explore; time spent in open versus closed areas were used to evaluate anxiety-related behavior. Mice that spent more time in the open area were considered to have less anxious behavior.
3. Y-Maze: Y-maze spontaneous alternation measures exploratory behavior based on the willingness of the mice to visit a new arm of the maze rather than a familiar arm. The Y-maze contains three arms at a 120-degree angle (44 cm length, 8 cm width, 20 cm height). Mice are placed in the center of the maze and given 5 min to explore. Arm entries were recorded and percent of spontaneous alternations was calculated. Arm entry was defined as all four limbs present within the arm. Mice had to make at least 10 arm entries to be used in the analysis.
4. Open Field Test: Mice were given 10 min a day for 3 consecutive days to freely explore an arena (40x40 cm). Distance traveled, velocity of movement within the arena, and time in center versus periphery were recorded and analyzed. Anxiety-related behavior is assessed by the duration spent in the center of the field versus the periphery. Mice that spend more time in the center than the periphery are considered to have decreased anxiety.
5. Novel Object Recognition: NOR tests reference memory. Mice are given 3 min to explore two identical objects. After a 30 min ITI, mice were given 5 min with one of the same objects (familiar) and a novel object in the place of one of the original objects. Total time spent investigating the individual objects during each testing phase was recorded. Increased time spent with the novel object versus the familiar object (an increase in recognition index) represents increased recognition memory.
6. Object Location Memory: OLM tests spatial memory and is performed similarly to NOR. First mice are given 3 min to explore two identical objects. After a 30 min ITI, one of the objects was moved to a different location within the arena and mice were given 5 min to explore. The time spent with the object that's in the new location compared to time spent exploring both objects is the discrimination index.
7. Barnes Maze: Barnes maze tests visual spatial learning and memory. The maze consists of a round arena (100 cm in diameter) that contains 20 holes (5 cm diameter) equally spaced around the perimeter (7.5 cm apart), one of which leads to an escape box. Mice were placed at the center of the arena under a box; once the box is removed the mice were given 3 min to find the escape box. Training consisted of two trials a day for six consecutive days. On the 7th day, the table was turned 180 degrees and the escape box was removed and a 5 min probe trial was done to test memory retention. Latency to find the escape hole, amount of time the mouse spent searching in the escape box quadrant, and number of incorrect holes visited (errors) were recorded. Two weeks after the probe trial, mice underwent a single trial (two-week retention) with the escape box intact. Time to find the escape box was recorded.

Supplementary Figures

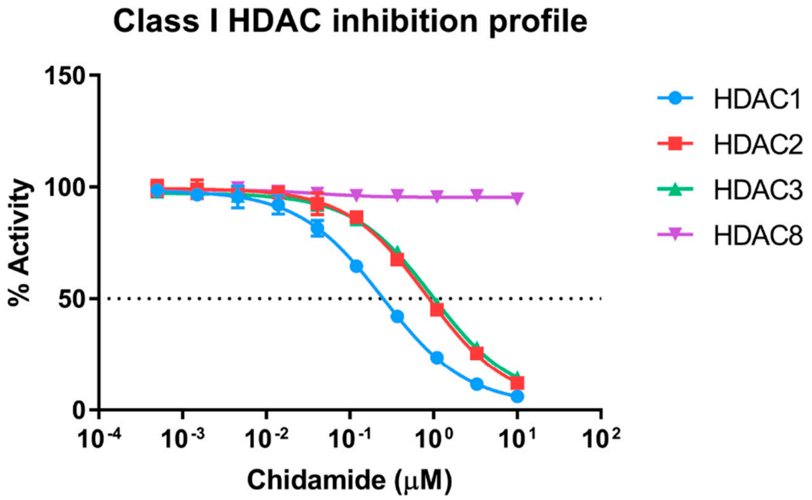

	IC$_{50}$ μM	
	**Chidamide**	**Reference**
**HDAC1**	0.25	0.049 (SAHA)
**HDAC2**	0.82	0.082 (SAHA)
**HDAC3/NCOR2**	1.0	0.052 (SAHA)
**HDAC8**	>10	1.3 (TSA)

Figure S1: IC50 for Chidamide.

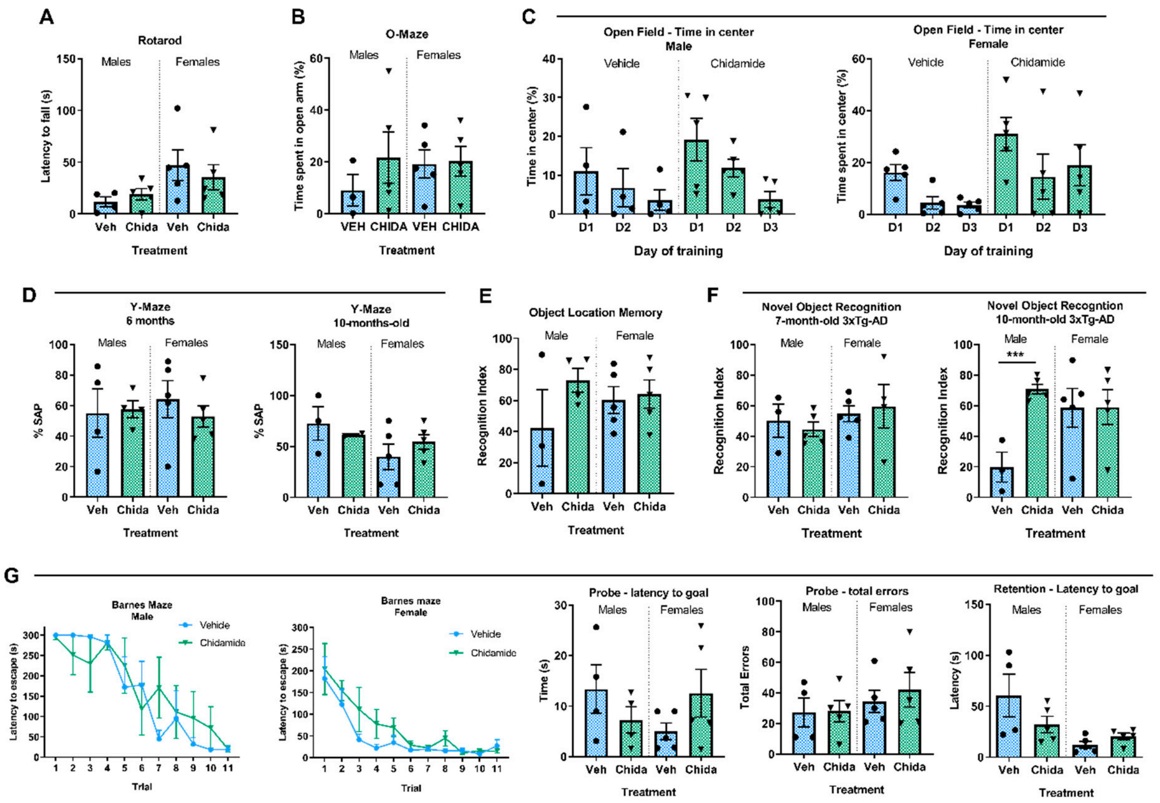

Figure S2: Sensorimotor, anxiety-related, and memory-related behavior. The graphs represent mean±SEM. Unpaired Student's *t* test or Mann-Whitney test was used for comparisons of two means. ***p<0.001; *n*=3-5/group.
